# In Vitro Wound Healing and Anticancer Effects of Ixora coccinea in Malignant Melanoma Cell Lines

**DOI:** 10.7759/cureus.58958

**Published:** 2024-04-24

**Authors:** Jasmin Sajini R, Vinodhini Chandrasekar, Chamundeeswari D, Karthik Rajendran, Anupma Jyoti Kindo, Jayakumari Swaminathan

**Affiliations:** 1 Pharmaceutical Chemistry, Sri Ramachandra Institute of Higher Education and Research, Chennai, IND; 2 Pharmacy, Sri Ramachandra Institute of Higher Education and Research, Chennai, IND; 3 Pharmacy, Meenakshi Academy of Higher Education and Research, Chennai, IND; 4 Bioanalytics and Analytics, Scitus Pharma Services Pvt. Ltd., Chennai, IND; 5 Microbiology, Sri Ramachandra Institute of Higher Education and Research, Chennai, IND; 6 Pharmacognosy, School of Pharmaceutical Sciences, Vels University, Chennai, IND

**Keywords:** 2vcj protein, human dermal fibroblasts, fluorescence microscope, apoptosis, dna damage

## Abstract

Background

*Ixora coccinea* is a medicinal plant with many active constituents that are responsible for wound healing and have anticancer properties. Herbal extracts increase the mechanisms related to wound healing, like blood clotting, fighting infection, and epithelialization. The effect responsible for this property may be the presence of phytoconstituents like flavonoids, polyphenols, and alkaloids. Many researchers have evaluated the wound-healing effect of *I. coccinea* leaf extract in aqueous methanol. This study aimed to determine the *in vitro* wound healing and anticancer efficacy of *I. coccinea *leaf ethyl acetate extract and evaluate the *in silico* docking of the selected phytoconstituents of *I. coccinea *in the 2vcj protein*.*

Materials and methods

The human dermal fibroblast cell line was used to determine the rates of cell migration and proliferation for evaluating the wound-healing effect of the *I. coccinea* leaf ethyl acetate fraction. 4',6-diamidino-2-phenylindole (DAPI) fluorescence labeling was used to estimate the rate of cell migration. The one-step TUNEL (TdT-mediated dUTP Nick-End Labeling) in situ apoptosis kit and the annexin V-FITC/7-AAD apoptosis kit were used to perform DNA damage assays in the malignant melanoma cell line. The ethyl acetate fraction of *I. coccinea* leaves was analyzed for its impact on wound healing markers, including keratin-10, keratin-14, type IV collagen, and α-SMA.

Results

The wound-healing nature was interesting in the ethyl acetate fraction at doses of 50 µg/mL and 100 µg/mL. Both studies involved in the DNA damage study against malignant melanoma cell lines showed the cleavage of apoptotic cancer cells, which was detected using a fluorescence microscope. When compared with the control, a dose of 100 μg/ml of ethyl acetate fraction from the leaves of *I. coccinea* showed fibroblast migration of cells into the wound area. The statistical values were considered significant at the level of *P* < 0.05. *An in silico* docking study on the 2vcj protein revealed that selected phytoconstituents of *I. coccinea *resulted in good docking scores to inhibit Hsp90.

Conclusion

*I. coccinea* ethyl acetate leaf extract can inhibit the growth of malignant melanoma cell lines and promote wound healing, as shown by the study results. It might be a viable therapeutic modality for skin cancer.

## Introduction

The process by which an intricate series of cellular and biochemical events combine to restore the strength and structural integrity of wounded tissues is known as wound healing [1]. In the olden days, it was evident that many people used plants to promote wound healing. Various in vitro and in vivo assays are available to prove wound healing using phytoconstituents and isolated compounds to influence the formation of new tissues [2]. The ethanolic extract of the root of *Ixora coccinea* has significant (P<0.001) wound-healing activity compared with standard drugs and shows good antifungal activity [3]. Using a circular excision paradigm, the promising *I. coccinea* methanol extract (IxME) was tested for its ability to promote wound healing in vivo in Wistar rats using seven-day postoperative wound granulation tissues, wound contraction measurements, hydroxyproline quantification, and western blotting for collagen type III (COL3A1), basic fibroblast growth factor (bFGF), and Smad-2, -3, -4, and -7 [4].

The hydrogel was made from dried* I. coccinea* leaves, and its antimicrobial activity was assessed using the agar-well method with *Pseudomonas aeruginosa*, *Staphylococcus aureus*, and *Staphylococcus epidermidis*. The results were compared with the standard medication, 2% w/v mupirocin ointment. The wound-healing properties of the preformulated hydrogel against povidone-iodine ointment were investigated using a wound excision model in rats [5]. The phytochemical, antioxidant, and antimicrobial effects of* I. coccinea* and *I. alba *were compared. Out of petroleum ether, chloroform, ethyl acetate, and hydroalcoholic extract (70:30, v/v), the ethyl acetate fraction of* I. coccinea* was reported to have high antioxidant and good antimicrobial effects. This may be due to the high flavonoid content of *I. coccinea *[6]. 

The anti-inflammatory potential of aqueous leaf extract (ALE) of* I. coccinea* by oral administration (500, 1000, and 1500 mg/kg) using carrageenan-induced paw edema proved that the early and late phases of the inflammatory response, as well as the edema that persisted between the two phases, were markedly compromised by ALE [7]. The presence of phenolics and isomers was observed in aqueous methanolic (70% methanol) extracts of *I. coccinea* leaves and stems by LC-MS. A C18 amide reverse-phase HPLC column allowed the separation of phenolic compounds, including different isomers. For the LC-MS measurements, the negative ion mode was used to obtain better tandem mass spectra and high-resolution mass spectra [8]. The effects of *I. coccinea* flower ethanol extract on different healing process parameters were demonstrated. Wet granuloma tissue weight showed a noteworthy increase [9].

The most common phytoconstituents present in *I. coccinea *were quercetin and rutin. Many studies have revealed the presence of these active constituents responsible for antioxidant and anticancer properties [10]. 2.8% of camptothecin was isolated from the young and mature leaves of* I. coccinea* [11]. One type of topoisomerase inhibitor is camptothecin (CPT). When M. E. Wall and M. C. Wani systematically screened natural materials for anticancer medicines in 1966, they made this discovery. It was separated from the bark and stem of the native Chinese tree known as the Happy Tree (*Camptotheca acuminata*), which is used in traditional Chinese medicine [12].

More recently, China has begun clinically using it to treat gastrointestinal cancers. In early clinical trials, CPT showed anticancer efficacy, particularly against malignancies of the breast, ovaries, colon, lung, and stomach [13]. Nevertheless, because of its poor solubility and documented side effects when used therapeutically, medicinal and synthetic chemists have created several syntheses of camptothecin and its derivatives in an attempt to maximize the chemical’s advantages, with largely positive outcomes. The four CPT analogs topotecan, irinotecan, belotecan, and trastuzumab deruxtecan have been approved and used in cancer chemotherapy. *Chonemorpha fragrans *is one of the other plants reported to contain camptothecin. The molecular formula of camptothecin is C_20_H_16_N_2_O_4_. Camptothecin produces DNA damage that leads to apoptosis by binding to the topoisomerase I and DNA complex to form a ternary complex, stabilizing it and preventing DNA religation [14].

A pyrrolo [3,4-β]-quinoline moiety (rings A, B, and C), a conjugated pyridone moiety (ring D), and one chiral center at position 20 within the alpha-hydroxy lactone ring with (S) configuration (the E-ring) comprise the planar pentacyclic ring structure of CPT. One of the key elements in topoisomerase inhibition is its planar structure [15]. One plant flavonoid belonging to the flavonoid group of polyphenols is quercetin. Similar to many other phenolic heterocyclic compounds, it has anti-inflammatory and antioxidant properties. Among the glycosylated variants are quercetin and rutin. It can be found in a wide variety of fruits, vegetables, grains, leaves, and seeds. Common foods that contain significant levels of it include red onions, capers, and kale [16]. It is used as a component in meals, drinks, and dietary supplements and has a bitter taste.

Along with the human QR1 homolog, quercetin is a selective inhibitor of quinone reductase 2 (QR2), an enzyme that catalyzes the metabolism of hazardous quinolines. In plasmodium, the inhibition of QR2 can induce fatal oxidative stress [17].

Rutin is a rutinoside that is essentially quercetin with glucose and rhamnose sugar groups in place of the hydroxy group at position C-3. It serves as both an antioxidant and a metabolite. It is a rutinoside, a tetrahydroxyflavone, a disaccharide derivative, and a quercetin O-glucoside. One of the phenolic chemicals present in *Carpobrotus edulis*, an invasive plant species, is rutin. Its name derives from the name of the plant, *Ruta graveolens*. *Aspergillus flavus* contains the enzyme quercitinase, which is involved in the catabolism of rutin [18]. 2vcj protein inhibitors of the Hsp90 molecular chaperone show considerable promise as potential chemotherapeutic agents for cancer [19].

## Materials and methods

Plant extract

The plant used for this study was *I. coccinea*. The leaf and flower parts were used for the study. The maceration method was used to extract the parts of the plant with petroleum ether, chloroform, ethyl acetate, and hydroalcohol (ethanol and water, 70:30 v/v) solvents. From the preliminary phytochemical antioxidant assay, the leaf ethyl acetate extract of *I. coccinea* was selected for the wound healing assay.

Previously, we established and published a comprehensive depiction of the extraction procedure, along with initial experiments aimed at identifying the optimal fraction of* I. coccinea* [6]. Consequently, the leaf ethyl acetate extract of *I. coccinea* was selected for the present study.

In vitro wound-healing assay

Two 24-well plates containing 12 specially treated plastic inserts each were used in the wound-healing assay with the human dermal fibroblast (HDF) cell line. The inserts generate a wound field with a specified gap of 0.9 mm, allowing the rates of cell migration and proliferation to be measured. Migratory cells can extend protrusions, which in turn can penetrate and seal the wound field. The rates of cell migration and proliferation were measured by manual fixation and microscopic imaging. At predetermined times, a fixing solution is provided to stop the cells. The outcomes were observed using fluorescence and light microscopy.

*I. coccinea* leaf ethyl acetate sample was tested in triplicate. A cell suspension was made in media containing 10% fetal bovine serum (FBS) at 0.5-1.0 x 106 cells/mL. The pipette tip was carefully inserted through the open end at the top of each well to add 500 µL of cell suspension. Two-hundred fifty µL of cell suspension was added to each side of the insert’s open ends for the best possible cell dispersion. Overnight, the cells were placed in a cell culture incubator. Slow aspiration was done, and then the media was removed from the wells. To remove debris and dead cells, the wells were washed with media. Lastly, wells were filled with media to maintain cell hydration. The wells were seen through a bright light microscope. If debris or detached cells remained in the wells, the wash was repeated. After washing, the cell culture and wound healing process was continued by adding media containing FBS and sample extract. A cell culture incubator was used to incubate the cells. Wound closure was monitored with imaging software. The percentage closure or migration rate of cells into the wound field was measured. Wound healing results were visualized using phase contrast and DAPI fluorescence labeling [20- 23].

DNA damage assay

DNA Damage Assay Using a One-Step TUNEL In Situ Apoptosis Kit

DNA damage assay was performed on cell lines of malignant melanoma. Certain DNA endonucleases were triggered during cell death and cleave genomic DNA between nucleosomes. Apoptotic cell DNA was broken up into multimers of 180-200 base pair pieces, which is the same as the oligonucleosomal size. As a result, on an agarose gel, apoptotic cells’ DNA usually migrates as a ladder of 180-200 bp. Terminal deoxynucleotidyl transferase (TdT) can catalyze the exposed 3′-OH of fragmented DNA using fluorescein-labeled dUTP, which can be observed under a fluorescence microscope.

After adding 100 μL of TdT equilibration buffer to the *I. coccinea* leaf ethyl acetate sample, the sample was incubated for 10-30 min at 37 °C. The liquid surrounding the sample regions was blotted using absorbent paper. Each slide received 50 μL of the labeling working solution and was incubated for 60 min at 37 °C under shaded light in a humidified environment. PBS was used to wash the slides three times for five minutes each. Using absorbent paper, the liquid surrounding the sample regions was carefully blotted. The addition of the DAPI working solution was followed by five minutes of RT incubation under shaded light. PBS was used four times on the slides, for five minutes each time. Using absorbent paper, the liquid surrounding the sample regions was carefully blotted. To seal the slides, an antifluorescence quenching agent was used [24,25].

DNA Damage Assay Using the Annexin V-FITC/7-AAD Apoptosis Kit

Annexin V is a member of the annexin family and binds to phosphatidylserine (PS) in a calcium-dependent manner. Annexin V-FITC, the FITC-conjugated format, binds specifically to PS on the outer leaflet of the apoptotic cell membrane and can be detected by fluorescence microscopy. Due to the loss of integrity of the membrane, 7-AAD can enter late apoptotic or necrotic cells to stain DNA. Cells at different apoptotic stages can be distinguished using annexin V and 7-AAD.

Induced apoptosis of suspension cells was performed using reagents of interest. Cell cultures were collected and centrifuged at 300 ×g for five minutes, and the supernatant was discarded. PBS was added to wash the cells. With the *I. coccinea* leaf ethyl acetate extract added, the cells were gently resuspended, followed by cell counting. The cell suspension was split into tubes, 1 to 5 × 105 cells for each, centrifuged at 300 ×g for 5 min, and the supernatant was discarded. PBS was added to wash the cells, and the supernatant was discarded. Five hundred μL of 1× annexin V binding buffer was added to resuspend the cells. Five μL of annexin V-FITC and 5 μL of 7-AAD were added to each tube. The cells were vortexed gently and incubated at room temperature for 15-20 minutes in the dark. The cells were analyzed immediately using the proper machine settings. Otherwise, the cells are placed on ice in the dark and analyzed within one hour. The samples were directly analyzed under a fluorescence microscope [26].

In silico docking studies of selected phytoconstituents on the 2vcj protein

The protein known as 2vcj is a key component linked to skin cancer, specifically identified as a 4,5-diaryl isoxazole heat shock protein 90 (Hsp90) chaperone. It is important to recognize that Hsp90 significantly contributes to the cellular environment of eukaryotic organisms. This molecular chaperone, widespread across various cell types, plays a crucial role in blocking apoptotic processes, essential for the survival of tumorigenic cells. Therefore, targeting Hsp90 inhibition has become a strategic focus in developing chemotherapeutic interventions, promising significant advances in the fight against cancer.

The critical role of Hsp90 in keeping tumor cells viable highlights the potential therapeutic value of Hsp90 inhibitors. Researchers design these compounds to selectively disrupt Hsp90's normal functioning, inducing cellular stress and apoptosis in tumor cells. Considering Hsp90's central role in controlling various signaling pathways crucial for tumor growth and survival, inhibiting it could result in a comprehensive blockage of oncogenic signaling, thereby hindering tumor progression [19].

Researchers identified and confirmed the presence of phytoconstituents, such as quercetin, camptothecin, and rutin, in the leaves of *I. coccinea* using LC-MS and HPLC techniques, as reported in studies [8,27-28]. This discovery highlights the plant's potential for yielding compounds of interest for further pharmacological studies.

In light of this, the current study embarked on an investigation to assess the efficacy of specific phytoconstituents against the 4,5-diarylisoxazole Hsp90 chaperone. Phytoconstituents, such as quercetin, camptothecin, and rutin being bioactive compounds derived from *I. coccinea* leaf, possess a wide range of biological activities, including anticancer properties. The rationale behind the selection of these compounds rests on their potential to bind to Hsp90, thereby neutralizing its function.

Preparation of the Receptors

The structural receptors of the 4,5 diaryl isoxazole Hsp 90 chaperone protein (PDB ID: 2VCJ, Resolution-2 Å) were obtained from the protein data bank, and molecular docking studies were performed. The selected target was prepared for docking using the AutoDock MG Tool and saved in the PDBQT file format. The protein was then prepared by removing the active ligand, protonating the structure with polar hydrogens, removing unbound water molecules, and minimizing the structure to a specified gradient level that is best suited for the prepared ligand [29].

Software

With a trial license for ChemDraw (PerkinElmer, Revvity, USA), the stated chemicals in the plant extracts, such as quercetin, campothecin, and rutin, were identified. AutoDock Vina was used to assist with computational investigations. Using a grid box, AutoGrid was used to prepare the grid map.

## Results

In vitro wound-healing assay

In an earlier published study [6], we identified that 100 μg of *I. coccinea* leaf ethyl acetate extract showed 86.30% inhibition in the A375 cell lines, and the IC_50_ value was reported as 7.96 μg/mL.

**Figure 1:**
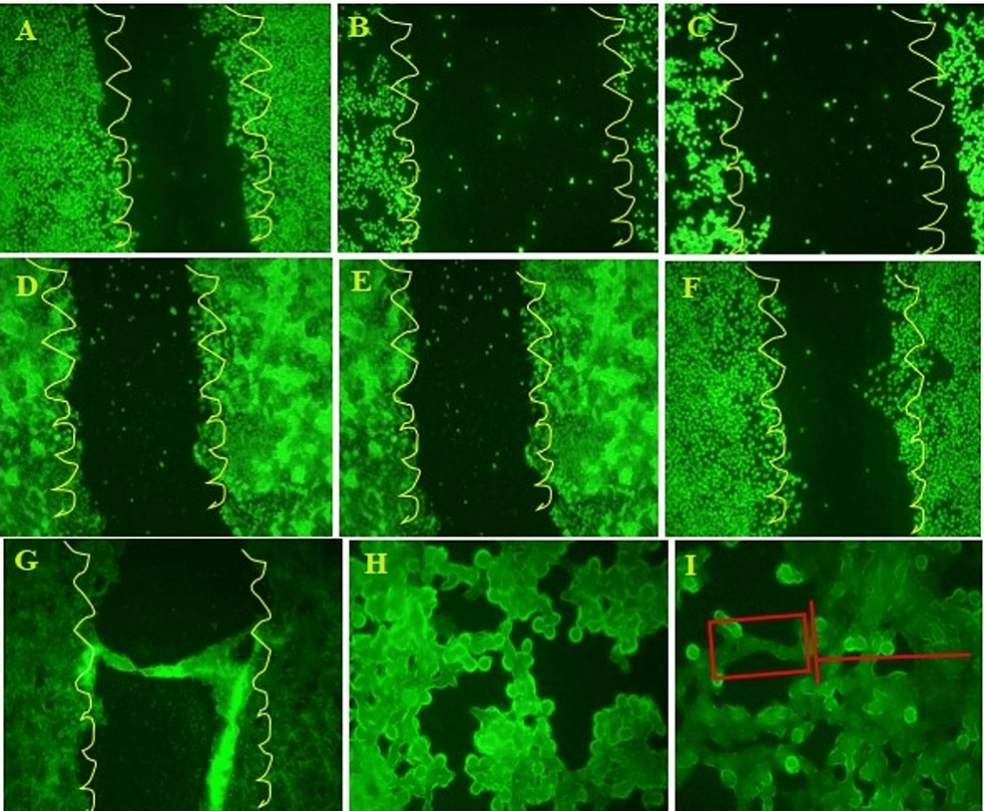
Photomicrograph representation of the in vitro scratch assay of Ixora coccinea leaf ethyl acetate extract at different concentrations at different time intervals. A: Control at 0 hour; B: magnified view of control at 24 hours; C: magnified view of control at 48 hours, D: *Ixora coccinea* leaf ethyl acetate extract 50 µg/mL at 24 hours; E: *Ixora coccinea* leaf ethyl acetate extract 50 µg/mL at 48 hours; F: *Ixora coccinea* leaf ethyl acetate extract 100 µg/mL at 24 hours; G: *Ixora coccinea* leaf ethyl acetate extract 100 µg/mL at 24 hours; H and I: magnified view of 100 µg/mL at 48 hours.

**Figure 2:**
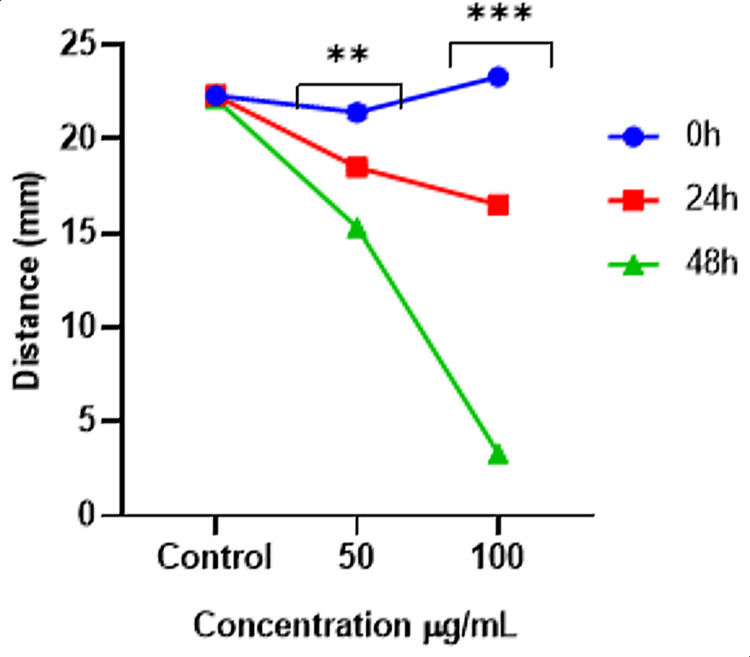
Wound closure (mm) by Ixora coccinea leaf ethyl acetate extract at 100 µg/mL at different time intervals.

Using the wound closure assay, the impact of the *I. coccinea *leaf ethyl acetate fraction on fibroblast migration was evaluated. At time intervals of 0 hours, 24 hours, and 48 hours, *I. coccinea* leaf ethyl acetate extract was used to observe wound healing assays with doses of 50 and 100 μg/mL, as depicted in Figure 1. Fibroblast migration proved the wound healing. The magnified view of the 100 μg/mL dose showed the fibroblast migration and wound healing properties of the *I. coccinea* leaf ethyl acetate fraction. The fibroblasts migrated at a distance measured in millimeters (mm), and the graph (Figure 2) was plotted between the distance of wound closure and the *I. coccinea* leaf ethyl extract, which was dosed at two different concentrations. One hundred μg/mL of *I. coccinea* leaf ethyl acetate extract developed fibroblast migration and showed the capability to heal the wound.

In an in vitro wound healing assay, the migration of HDFs to wound regions was stimulated by the *I. coccinea* leaf ethyl acetate fraction. Following the scratching of a confluent monolayer, the migration of cells into the denuded area was much higher in the presence of 50 µg/mL and 100 µg/mL *I. coccinea* leaf ethyl acetate fractions than in the unreacted control group after 24 and 48 hours. The outcome revealed that the *I. coccinea* leaf ethyl acetate fraction significantly increased fibroblast cell migration and proliferation. The statistical values were considered significant at the level of P < 0.05.

**Figure 3:**
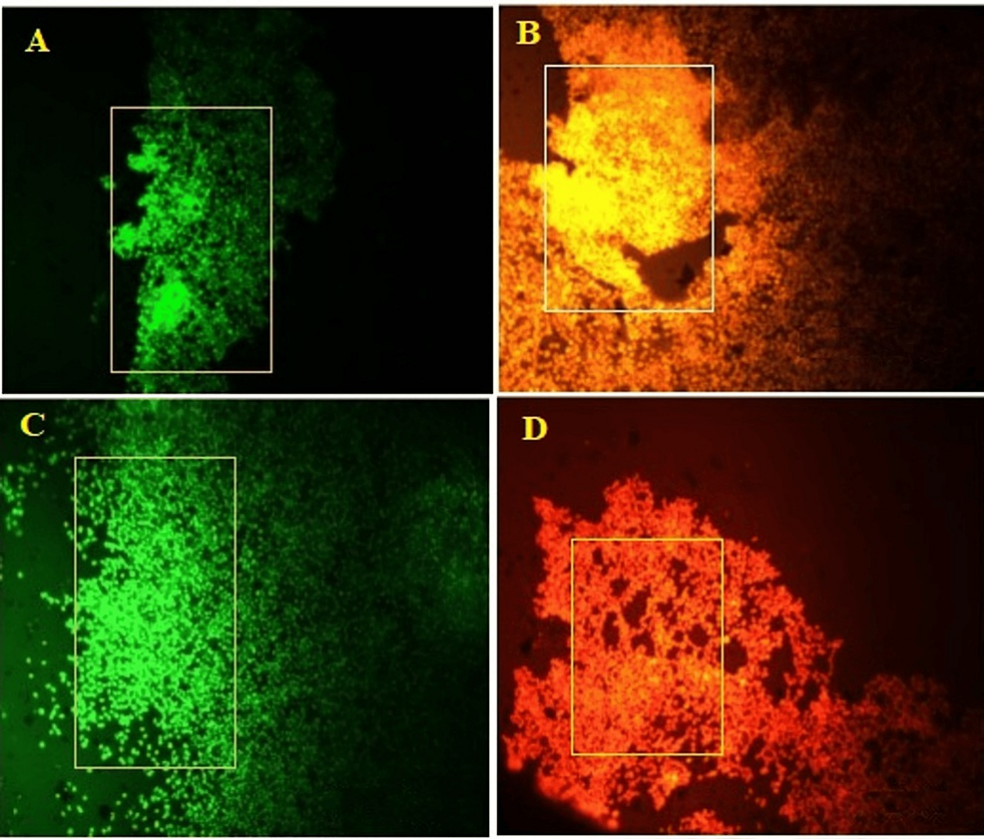
Expression of wound healing markers in human dermal fibroblast cell lines by Ixora coccinea leaf ethyl acetate extract at 100 µg/mL. A: Keratin 10 marker; B: Keratin 14 marker; C: Type IV collagen marker; D: α- SMA marker.

*I. coccinea* leaf ethyl acetate extract was analyzed for its effect on skin markers, including keratin 10, keratin 14, type IV collagen, and α-SMA. After incubation with 4′,6-diamidino-2-phenylindole (DAPI, Sigma), we examined the nuclear staining. Figure 3 displays the healing property imaged using fluorescence microscopy.

DNA damage assay

**Figure 4:**
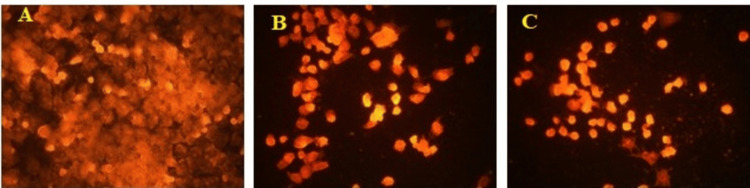
Apoptosis detected by one-step TUNEL in situ apoptosis kit in malignant melanoma cell lines using Ixora coccinea leaf ethyl acetate extract at different concentrations. A: control; B:* Ixora coccinea* leaf ethyl acetate extract 50 µg/mL; C: *Ixora coccinea* leaf ethyl acetate extract 100 µg/mL.

**Figure 5:**
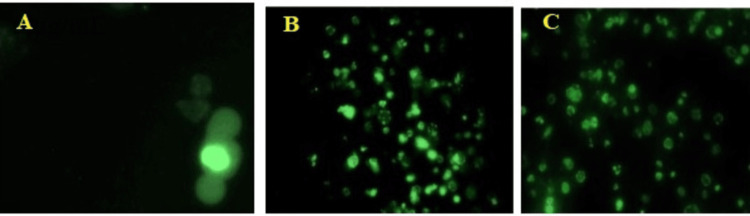
Apoptosis detected by Annexin V-FFITC/7-AAD in malignant melanoma cell lines using Ixora coccinea leaf ethyl acetate extract at different concentrations. A: control; B: *Ixora coccinea* leaf ethyl acetate extract 50 µg/mL; C: *Ixora coccinea* leaf ethyl acetate extract 100 µg/mL.

In a DNA damage assay, the effect of *I. coccinea *leaf ethyl acetate extract at 50 and 100 µg/mL doses on early and late apoptosis by one-step TUNEL in situ apoptosis kit and annexin V-FITC/7-AAD was examined to understand the basic mechanism of cell induction. At a dose of *I. coccinea* leaf ethyl acetate extract of 100 µg/mL, malignant melanoma cells are damaged and undergo apoptosis in the one-step TUNEL in situ Apoptosis Kit assay, as demonstrated in Figure 4. Figure 5 shows that the annexin V-FFITC/7-AAD study induced apoptosis in *I. coccinea* leaf ethyl acetate extract at a dose of 100 µg/mL. *I. coccinea* leaf ethyl acetate extract, as demonstrated by fluorescent microscopy examination using both apoptosis kits, has the capability to damage malignant melanoma cell lines and induce cleavage of apoptotic cancer cells.

In silico docking studies with the 2vcj protein

**Figure 6:**
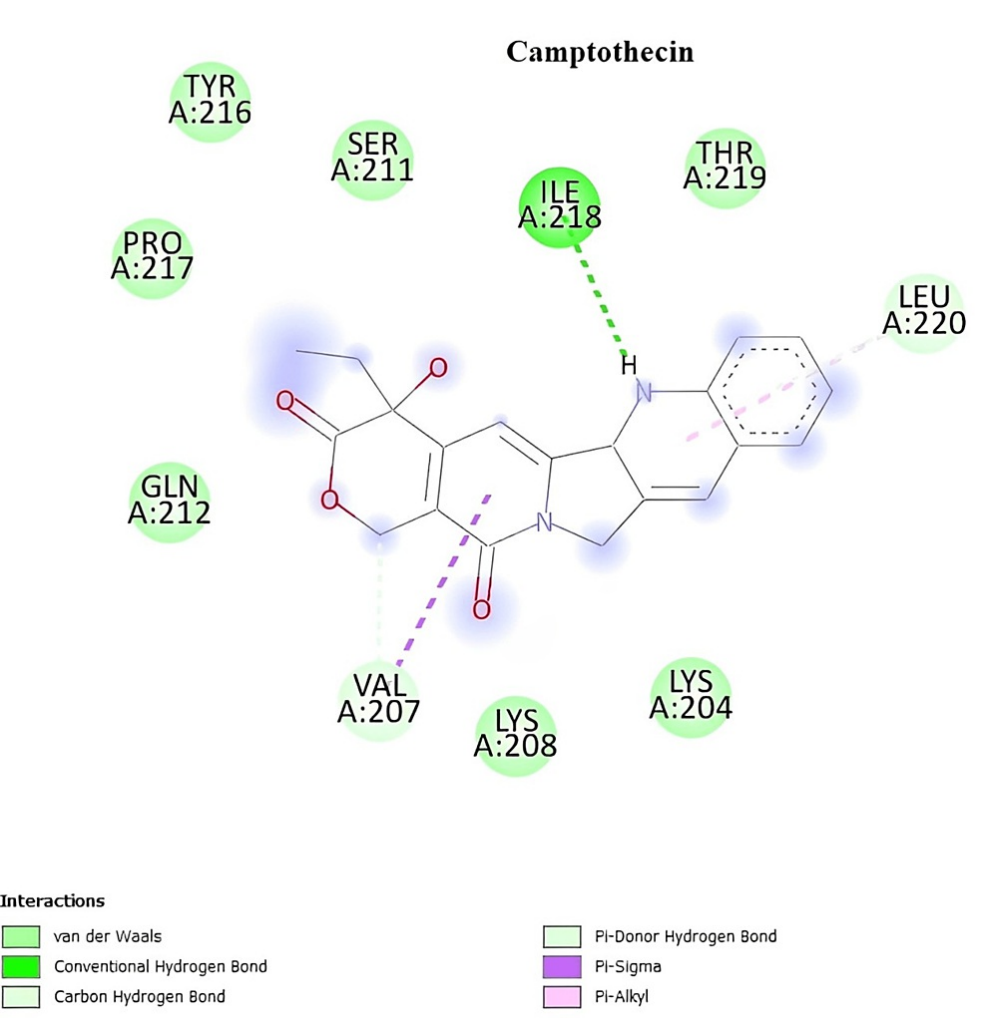
Two-dimensional interactions of camptothecin with the 2vcj protein.

**Figure 7:**
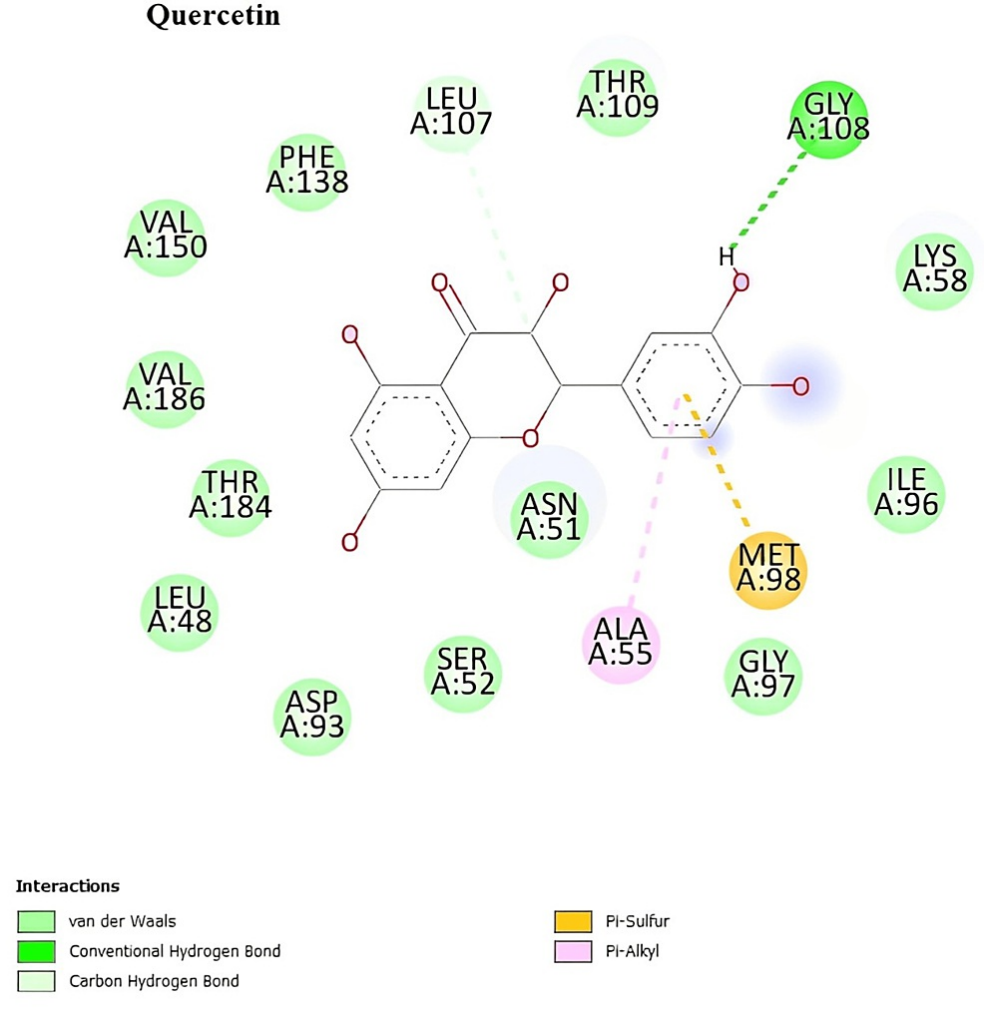
Two-dimensional interactions of quercetin with the 2vcj protein.

**Figure 8:**
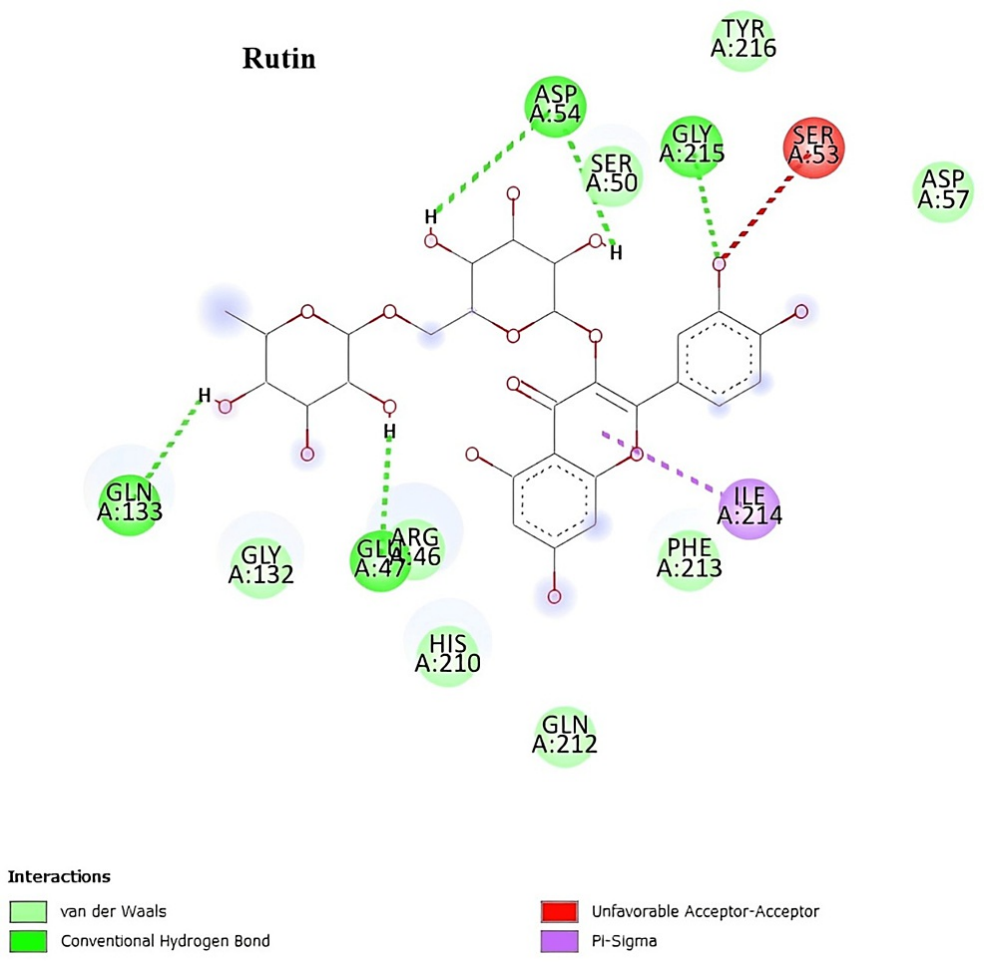
Two-dimensional interactions of rutin with the 2vcj protein.

The two-dimensional interactions of camptothecin showed the amino acid residues involved in the molecular interaction with the ligands, which are ILE A:218, LEU A:220, and VAL A:207 (Figure 6). The two-dimensional interactions of quercetin showed the amino acid residues involved in the molecular interaction with the ligands, which are GLY A:108, MET A:98, and ALA A:55 (Figure 7). The two-dimensional interactions of rutin showed the amino acid residues involved in the molecular interaction with the ligands, which are ASP A:54, SER A:50, GLY A:215, SER A:53, ILE A:214, GLU A:47, and GLN A:133 (Figure 8).

**Figure 9:**
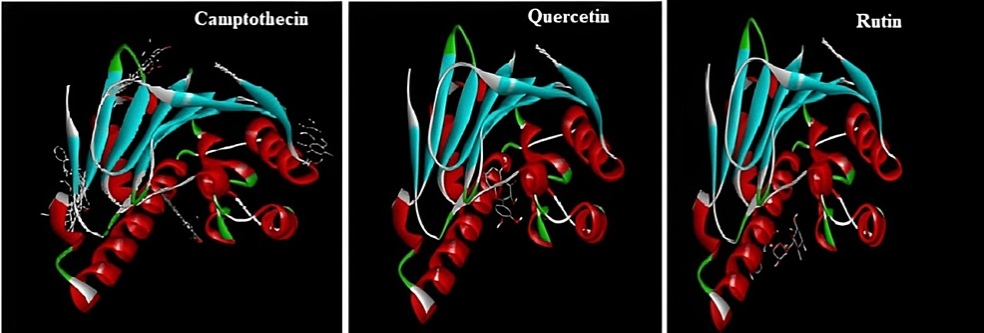
Three-dimensional interactions of camptothecin, quercetin, and rutin with the 2vcj protein.

The three-dimensional interactive image gives justification for the good interaction of selected phytoconstituents of Ixora coccinea with the 2vcj protein, which ultimately proves the anticancer potential of active constituents present in *I. coccinea* (Figure 9). The 2vcj protein binds camptothecin, quercetin, and rutin with the represented binding energies in Table 1. Rutin exhibits a higher binding affinity (-7.9) than quercetin (-7.6) and camptothecin (-6.9).

**Table 1:** Binding affinity of the selected phytoconstituents from Ixora coccinea with the 2vcj protein. The predicted binding affinity is expressed in kcal/mol (energy). RMSD u.b: root mean square deviation upper bound; RMSD l.b: root mean square deviation lower bound.

Binding Affinity	RMSD u. b	RMSD l. b	Binding Affinity	RMSD u. b	RMSD l. b	Binding Affinity	RMSD u. b	RMSD l. b
Camptothecin			Quercetin			Rutin		
-6.9	0	0	-7.6	0	0	-7.9	0	0
-6.7	37.207	34.796	-7.4	6.797	2.278	-7.9	6.726	2.016
-6.7	23.397	20.411	-7.4	1.436	1.194	-7.9	7.615	3.126
-6.5	5.714	3.385	-7.1	6.338	1.537	-7.8	10.699	6.252
-6.3	31.959	29.611	-6.9	5.006	2.934	-7.7	5.262	2.605
-6.3	34.746	32.53	-6.9	3.819	2.203	-7.5	8.633	2.848
-6.3	17.013	14.872	-6.8	7.314	5.782	-7.5	5.695	2.477
-6.3	31.487	28.879	-6.4	17.427	15.401	-7.5	16.196	11.998
-6.2	31.118	28.392	-6.4	6.147	4.424	-7.4	6.908	2.524

Numerous cancerous behaviors, including angiogenesis, antiapoptosis, metastasis, proliferation, and treatment resistance, are attributed to Hsp90. Tumors that exhibit multiple malignant behaviors may be simultaneously reversed by inhibiting Hsp90. Hsp90 inhibitors are excellent candidates for anticancer treatments. The molecular docking study aids in the computational prediction of the anticancer qualities of the extracts. The selected phytoconstituents present in the *I. coccinea* leaf exhibited high docking scores. As a result, it can inhibit and support the continuation of tumor cell proliferation. The docking study findings support the phytoconstituent affinity for proteins, suggesting that the extract of *I. coccinea *leaf can prevent cancer.

## Discussion

This study aims to investigate and expand upon the findings of an earlier study we published. The ethyl acetate fraction of *I. coccinea* leaves contained diterpene, flavonoids, phytosterols, and alkaloids, according to the phytochemical analysis of the previous study. The high-performance thin-layer chromatography (HPTLC) analysis proved the presence of quercetin. In the preliminary screening of the antioxidant study, the antimicrobial activity, minimum inhibitory concentration (MIC), total flavonoid content, and in vitro anticancer evaluation of the *I. coccinea* leaf in different extraction solvents were assessed. The results indicated that *I. coccinea* leaf ethyl acetate has a potential fraction that inhibits the growth of A375 cell lines, which are malignant melanoma cell lines [6]. This study aims not only to strengthen previous studies but also to explore new aspects and application possibilities of *I. coccinea*.

*I. coccinea* leaf ethyl acetate showed wound-healing effects in our study, as evidenced by the migration of a human dermal fibroblast cell line. DNA repair is essential for preventing tumor development. DNA repair mechanisms protect against cancer by preventing mutations. Once cancer has developed, DNA damage analysis is used to determine the inhibition of cancerous growth and the induction of apoptotic cell death. The selected potent fraction damaged the malignant melanoma cancer cell line, as shown by the DNA damage assay.

In the in silico docking study, campothecin, quercetin, and rutin were shown to interact with and prove the effect on the 2Vcj protein for skin cancer. The selected plant components target this molecular chaperone and inhibit the signaling cascade that stimulates tumor growth, thereby disrupting the ability of Hsp90 to maintain tumor cell survival and proliferation. This strategy opens new avenues for cancer therapy development by leveraging the unique biological activities of plant components to address the complex disease processes that contribute to cancer development.

In previous studies, the ethanolic root extract of *I. coccinea* had wound healing and antibacterial activity [3]. The methanolic leaf extract of *I. coccinea* evidenced wound contraction, higher hydroxyproline content, and improved the histopathology of granulation tissue. In addition, topical application of *I. coccinea* methanol extract stimulated fibroblast growth factor [4].

Pretreatment of HepG2 cells with aqueous extract of *I. coccinea* flowers significantly decreased the amount of reactive oxygen species, mitochondrial membrane potential, apoptosis, and DNA damage at p-value < 0.01 [30].

In silico antidiabetic activity of quercetin, biochanin A, and β-amyrin from the ethanolic flower extract of *I. coccinea* Linn docked with insulin receptor proteins [31]. Nine selected plant secondary metabolites from *I. coccinea*, *Mimosa pudica*, and *Origanum vulgare* in the Philippines were molecularly docked using AutoDock simulation software and Biovia Discovery Studio against the RND efflux pump system, AcrAB-TolC, of *Escherichia coli*. All the selected metabolites have negative binding energies, implying high ligand-receptor affinity and good stability, especially the secondary metabolites of I. coccinea. Metabolites that have remarkable properties similar to the existing efflux pump inhibitors include lupeol, quercetin, galangin, kaempferol, and ursolic acid [32].

Twenty compounds scored binding affinities of −11.2 to −8.1 kcal/mol toward PEX9 as top hits after screening 200 compounds. *I. coccinea* plants have been thoroughly investigated, and the hits were found to be linked to them as they were isolated from them [33]. Ixoratannin A-2 and other constituents of *I. coccinea* strongly bind the multidrug-resistant HIV protease enzyme, CXCR4, and the human elongin C protein [34].

Limitation

The present study showed a positive response in wound healing and the capability to damage malignant melanoma cells with the aid of *I. coccinea* leaf ethyl acetate extract for treating skin cancer. Further topical formulation and its evaluation, in vivo wound healing, anticancer effects, dermal toxicity, and clinical studies may provide clear evidence for the use of the formulation containing the *I. coccinea* potential fraction for treating skin cancer.

## Conclusions

From the preliminary evaluation, it was found that the ethyl acetate fraction of *I. coccinea* leaf was reported to have high flavonoid content and antioxidant potential with a lower IC_50_ value, so the ethyl acetate fraction of the leaf was selected to evaluate the wound healing assay in the present study. It was proven that the migration of HDFs to wound regions was stimulated by the *I. coccinea* leaf ethyl acetate fraction. DNA damage assay results indicate that the active fraction is capable of cleaving apoptotic melanoma cancer cells. In silico docking studies revealed that camptothecin, quercetin, and rutin have good docking scores and binding abilities with the skin cancer protein 2vcj. Because of the poor solubility of phytoconstituents of *I. coccinea* by oral route, they can be effectively administered by topical route and attain good bioavailability. Hence, this study gives a strong idea to proceed with research with the selected *I. coccinea* topical formulations for the treatment of skin cancer.
